# Comprehensively prognostic and immunological analyses of GLP-1 signaling-related genes in pan-cancer and validation in colorectal cancer

**DOI:** 10.3389/fphar.2024.1387243

**Published:** 2024-07-22

**Authors:** Chaojun Zhu, Yihong Lai, Chengdong Liu, Lan Teng, Yuxin Zhu, Xinyu Lin, Xinyi Fu, Qiuhua Lai, Side Liu, Xiaohan Zhou, Yuxin Fang

**Affiliations:** ^1^ Guangdong Provincial Key Laboratory of Gastroenterology, Department of Gastroenterology, Nanfang Hospital, Southern Medical University, Guangzhou, China; ^2^ Department of Infectious Diseases, Nanfang Hospital, Southern Medical University, Guangzhou, China; ^3^ Department of Gastroenterology, Zhuhai People’s Hospital (Zhuhai Hospital Affiliated with Jinan University), Zhuhai, China; ^4^ Department of Radiation Oncology, Nanfang Hospital, Southern Medical University, Guangzhou, China

**Keywords:** GLP-1, pan-cancer, prognosis, immune infiltration, immunotherapy

## Abstract

**Background:** Glucagon-like peptide-1 (GLP-1) has crucial impact on glycemic control and weight loss physiologically. GLP-1 receptor agonists have been approved for treatment of diabetes and obesity. Emerging evidence suggests that GLP-1 receptor agonists exert anticancer effect in tumorigenesis and development. However, the role and mechanism of GLP-1 signaling-related genes in pan-cancer still need further study.

**Methods:** We comprehensively investigated the aberrant expression and genetic alterations of GLP-1 signaling-related genes in 33 cancer types. Next, GLP-1 signaling score of each patient in The Cancer Genome Atlas were established by the single-sample gene set enrichment analysis. In addition, we explored the association of GLP-1 signaling score with prognostic significance and immune characteristics. Furthermore, qRT-PCR and immunohistochemistry staining were applied to verify the expression profiling of GLP-1 signaling-related genes in colorectal cancer (CRC) tissues. Wound-healing assays and migration assays were carried out to validate the role of GLP-1 receptor agonist in CRC cell lines.

**Results:** The expression profiling of GLP-1 signaling-related genes is commonly altered in pan-cancer. The score was decreased in cancer tissues compared with normal tissues and the lower expression score was associated with worse survival in most of cancer types. Notably, GLP-1 signaling score was strongly correlated with immune cell infiltration, including T cells, neutrophils, dendritic cells and macrophages. In addition, GLP-1 signaling score exhibited close association with tumor mutation burden, microsatellite instability and immunotherapy response in patients with cancer. Moreover, we found that the expression of GLP-1 signaling-related genes ITPR1 and ADCY5 were significantly reduced in CRC tissues, and GLP-1 receptor agonist semaglutide impaired the migration capacity of CRC cells, indicating its protective role.

**Conclusion:** This study provided a preliminary understanding of the GLP-1 signaling-related genes in pan-cancer, showing the prognosis significance and potential immunotherapeutic values in most cancer types, and verified the potential anticancer effect of GLP-1 receptor agonist in CRC.

## 1 Introduction

Obesity has become a threat to human health worldwide, with the rate of obesity more than 20% in some areas (Ruiz-Ojeda et al., 2016). Numerous evidences indicate that obesity leads to systemic chronic inflammation and is strongly associated with type 2 diabetes, cardiovascular disease, non-alcoholic fatty liver disease, along with many types of cancers (Lega and Lipscombe, 2020; Rohm et al., 2022).

Glucagon-like peptide-1 (GLP-1), a peptide hormone derived from proglucagon, is generated in the gut and brain stem primarily through the action of prohormone convertase 1/3 (Holst, 2007). GLP-1 is secreted by intestinal L cells in response to food digestion and functions by binding to GLP-1 receptor, which targets multiple organs physiologically. One of the essential roles of GLP-1 is the enhancement of insulin secretion by islet beta cells, which is glucose concentration dependent and it does not occur when plasma glucose concentration is normal (Gribble and Reimann, 2021). Moreover, GLP-1 also has physiological effects on losing weight, protecting pancreatic β cells, inhibiting glucagon secretion and suppressing gastric emptying, along with cardioprotective and neuroprotective effects (Hira et al., 2020; Brown et al., 2021).

However, GLP-1 is rapidly decomposed by dipeptidyl peptidase 4 after secretion under physiological conditions (Holst, 2007), which limits its clinical application. In this context, GLP-1 receptor agonists were developed. Compared with GLP-1, the latter has similar effect but different structure, thus its action time prolongs. Due to the excellent performance in blood glucose control and body weight reduction (Tamborlane et al., 2019; Nadolsky and Agarwal, 2021; Rubino et al., 2022), GLP-1 receptor agonists have been widely used in clinical practice. Subsequently, the potential roles of GLP-1 receptor agonists in neoplastic diseases also have been come into focus.

A previous study showed that PRKAR2B promoted epithelial-mesenchymal transition (EMT) and metastasis via Wnt/β-catenin pathway in prostate cancer, indicating the malignant potential of GLP-1 signaling-related genes (Sha et al., 2018). Moreover, earlier animal studies and drug clinical trials have suggested that the use of GLP-1 receptor agonists may increase the risk of pancreatitis, pancreatic cancer, medullary thyroid cancer, and breast cancer (Nachnani et al., 2010; Elashoff et al., 2011; Hashimoto Takigami et al., 2021). Nevertheless, subsequent meta-analyses and randomized controlled clinical trials did not find an association between GLP-1 receptor agonists and an increased risk of the above cancers (Ryder, 2013; Monami et al., 2017; Cao et al., 2019). In addition, a recent case-control study identifies the potential anticancer effect of GLP-1 receptor agonist exendin-4 on patients with both type 2 diabetes and cervical cancer (Mao D. et al., 2021). Moreover, GLP-1 receptor agonist liraglutide can reduce malignant biological behavior of colorectal cancer cells by inhibiting the PI3K/Akt/mTOR signaling pathway (Tong et al., 2022).

These studies vary from each other and only focus on the role of GLP-1 receptor agonists in one or some specific malignancies. Furthermore, nearly no researches have investigated the association between GLP-1 signaling-related genes and prognostic and immune landscape in pan-cancer. Therefore, we conducted an exploratory analysis of the expression and potential function of GLP-1 signaling-related genes in pan-cancer based on bioinformatics analysis and validated in colorectal cancer (CRC) by experiments *in vitro*.

## 2 Materials and methods

### 2.1 Data collection

The expression profiles and clinical data of The Cancer Genome Atlas (TCGA) and Genotype-Tissue Expression (GTEx) were collected from the UCSC Xena database (https://xenabrowser.net/datapages/). The list of 33 cancer types is presented in Supplementary Table S1. A total of 42 genes related to GLP-1 signaling were obtained from Gene Set Enrichment Analysis (GSEA) Molecular Signatures Database (https://www.gsea-msigdb.org/gsea/index.jsp) and the protein-protein interaction network of the genes was analyzed in STRING database (http://string-db.org) (Supplementary Figure S1). The list of 42 genes is presented in Supplementary Table S2.

### 2.2 Identification of differentially expressed genes

To identify differentially expressed genes related to GLP-1 signaling, data of tumor samples (from TCGA database) and normal tissue samples (from TCGA and GTEx databases) were analyzed using the “limma” R package, with the cut-off value of |log2-fold change| ≥ 1 and FDR <0.05. Next, the results were visualized as a heatmap using the “ggplot2” and “reshape2” R packages. Pearson correlation coefficient was used to explore the correlations among different genes related to GLP-1 signaling, and the results were represented as a heatmap.

### 2.3 Genetic mutation, DNA methylation and copy number variation analysis

The alterations (genetic mutation, DNA methylation and copy number variation (CNV)) analyses of genes related to GLP-1 signaling were carried out using Gene Set Cancer Analysis (GSCA, http://bioinfo.life.hust.edu.cn/GSCA). Student’s t-test was used to calculated the methylation difference among tumor and normal samples in each cancer type. The CNV summary includes homozygous amplification, homozygous deletion, heterozygous amplification and heterozygous deletion, which represents genetic amplification and deletion that occur on only one chromosome or a pair of chromosomes.

### 2.4 GLP-1 signaling score analysis

The GLP-1 signaling score of each patient in the TCGA cohort was estimated by the single-sample gene set enrichment analysis (ssGSEA) function of “GSVA” R package (Qin et al., 2023). By using the “limma” R package, we explored the differences in GLP-1 signaling score between paired normal and tumor tissues, along with the score across clinical stages of pan-cancer in TCGA database.

### 2.5 Prognostic analysis

Kaplan-Meier analyses were performed to explore the relationship between GLP-1 signaling score and overall survival (OS) of patients in TCGA database. In addition, the R packages “survminer” and “survival” were used to perform univariate Cox regression analyses and estimate the prognostic value of GLP-1 signaling score, including OS, disease-specific survival (DSS), disease-free interval (DFI), progression-free interval (PFI).

### 2.6 Tumor microenvironment (TME) and immune feature analysis

To investigate the association of GLP-1 signaling score and TME, we estimated the stromal score, immune score and tumor purity of each patient in TCGA using R package “ESTIMATE”. Then the correlations of GLP-1 signaling score with these TME scores were analyzed. Furthermore, we downloaded and analyzed the immune cell infiltration score of samples in TCGA from the ImmuCellAI database (http://bioinfo.life.hust.edu.cn/ImmuCellAI#!/) and TIMER2 database (http://timer.cistrome.org/). In addition, we analyzed the correlation of GLP-1 signaling score with immune-activating genes, major histocompatibility complex (MHC) genes, chemokines and chemokine receptors (Zhou et al., 2021).

To explore the effect of GLP-1 signaling score on the survival prognosis in patients who have undergone immunotherapy, we analyzed the data from three Gene Expression Omnibus (GEO, https://www.ncbi.nlm.nih.gov/gds) datasets (GSE32894: urothelial carcinoma; GSE61676: non-squamous non-small cell lung cancer; GSE135222: non-small cell lung carcinoma).

### 2.7 Human samples and cell lines

Human tissue specimens were collected from the Department of General Surgery, Nanfang Hospital, Southern Medical University, and all patients were pathologically diagnosed with colorectal cancer. This study was approved by the Ethics Committee of Nanfang Hospital. Human colorectal cancer cell lines (RKO, SW480, HCT116, SW620, HT29, LoVo and CaCO2), normal colorectal epithelial cells (FHC) and mouse colorectal cancer cells (MC38) were cultured in DMEM supplemented with 10% FBS.

### 2.8 RNA extraction and qRT-PCR

Total RNA was extracted from human samples and cells with Trizol reagent (TaKaRa, Japan). Hifair III 1st Strand cDNA Synthesis SuperMix (Yeasen, Shanghai, China) and SYBR Green Primix Pro Taq HS (Accurate Biology, Changsha, China) were used to obtain cDNA and quantitation. Relative mRNA level normalized to the control was analyzed with the 2^−ΔΔCT^ method. Primers used in this study are listed in the Supplementary Table S3.

### 2.9 Western blot and immunohistochemistry (IHC) staining

For Western blot assay, protein samples were separated by 10% SDS-PAGE and transferred to polyvinylidene fluoride membranes, which were incubated with an anti-ITPR1 antibody (1:1,000, 19962-1-AP, Proteintech, Wuhan, China), anti-ADCY5 antibody (1:500, 30153-1-AP, Proteintech) or anti-GAPDH antibody (1:20,000, 60004-1-Ig, Proteintech). The membranes were further incubated with a secondary antibody (goat anti-rabbit or anti-mouse IgG, 1:1,000, Beyotime, Shanghai, China) and visualized by image analysis system.

IHC staining was performed according to the instruction for IHC kit (PV-6001, ZSGB-BIO Beijing, China). The following primary antibodies were used: ITPR1 (1:250, 19962-1-AP, Proteintech), ADCY5 (1:250, 30153-1-AP, Proteintech).

### 2.10 Wound-healing assay and migration assay

For wound-healing assay, 4.5×10^5^ SW480 and MC38 cells were seeded in 6-well plate. When the cell confluency reached about 80%, wound injury was made with the tip of a sterile micropipette and images were taken by microscope. Migration assay was performed using a 24-well Transwell insert (8-mm-pore size) and 3×10^4^ SW480 cells or 4 × 10^4^ MC38 cells were suspended in serum-free medium in upper chamber, as well as medium containing 10% FBS in the lower chamber. The migration cells were fixed with 4% paraformaldehyde, stained and photographed.

### 2.11 Statistical analysis

Statistical analyses were performed using R software (version: 4.0.5). Data are presented as means ± standard error (SD). Differences between groups were analyzed using the Student’s t-test (two-tailed). Pearson correlation coefficient was used in all correlation analyses and the log-rank test was applied for Kaplan–Meier survival analyses. *P* < 0.05 was considered statistically significant and indicated as follow: *p < 0.05, **p < 0.01, ***p < 0.001, ****p < 0.0001; ns represents not significant.

## 3 Results

### 3.1 Aberrant expression of GLP-1 signaling-related genes in pan-cancer

**FIGURE 1:**
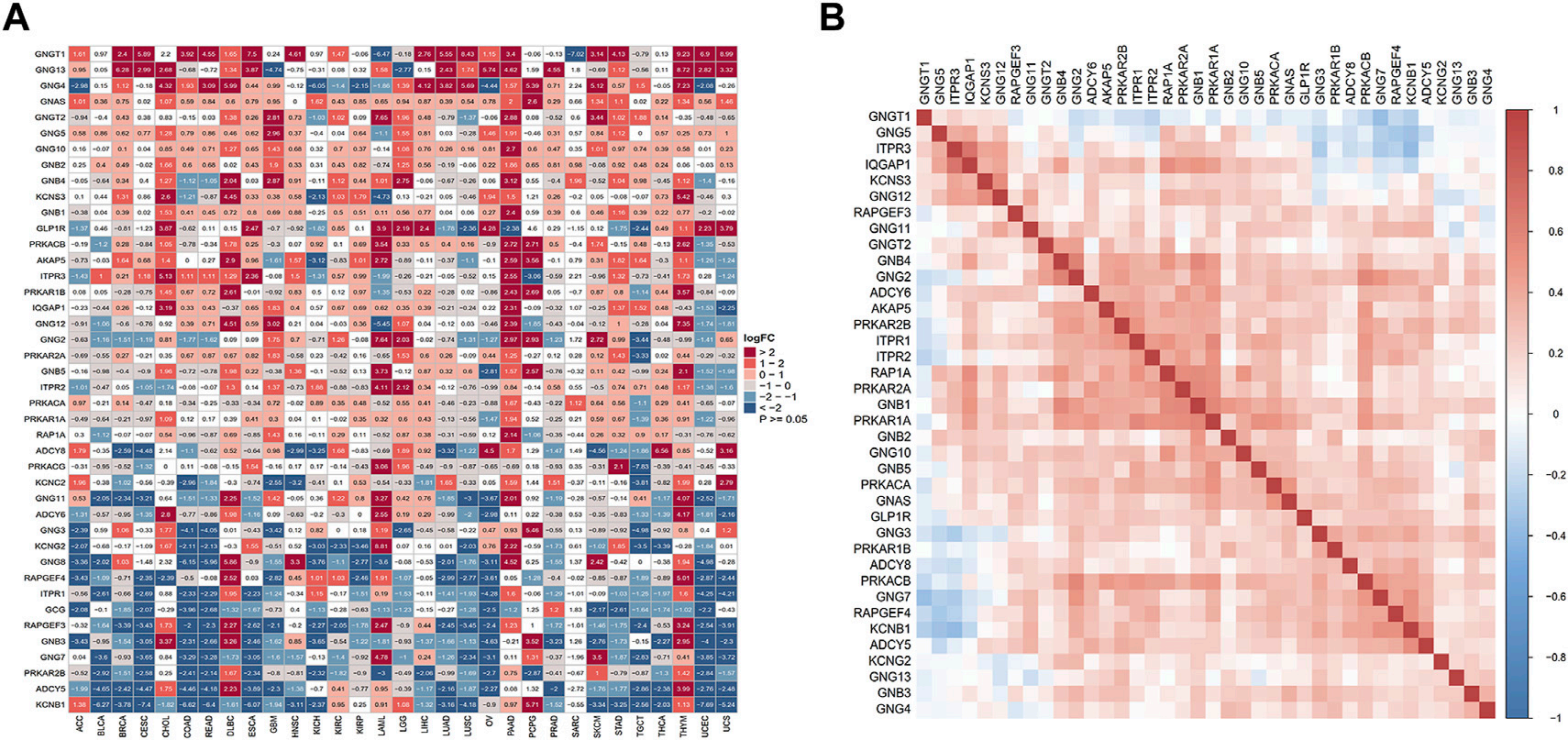
Aberrant expression of GLP-1 signaling-related genes in pan-cancer. **(A)** Differential expression analysis for genes related to GLP-1 signaling in 31 types of normal and tumor tissues from TCGA and GTEx databases. Color grid indicated *p* < 0.05 while white grid indicated *p* > 0.05. FC: fold change. **(B)** Association among GLP-1 signaling-related genes based on TCGA data. The gradation of color represents Pearson correlation coefficient.

To explore the expression profile of 42 genes related to GLP-1 signaling in pan-cancer, we performed differential expression analysis among 31 types of tumor and normal tissues in TCGA and GTEx datasets and found that all the genes were abnormally expressed in at least one type of cancer (Figure 1A). Specifically, most of the genes tended to be upregulated in PAAD, THYM and DLBC, while downregulated in CESC and UCEC. And some genes exhibited a consistent variation of expression level in various tumor types. GNGT1, GNG13 and GNG4 were significantly upregulated in most cancer types, while KCNB1, ADCY5, PRKAR2B, GNG7, GNB3, RAPGEF3, GCG, ITPR1 were downregulated in most cancer types (|log2-fold change| ≥ 1, *p* < 0.05). To analyze the potential association among these genes based on TCGA pan-cancer data, Pearson correlation coefficient was calculated and a positive correlation was found in most of the genes (Figure 1B).

### 3.2 Genetic alteration landscape of GLP-1 signaling-related genes

**FIGURE 2:**
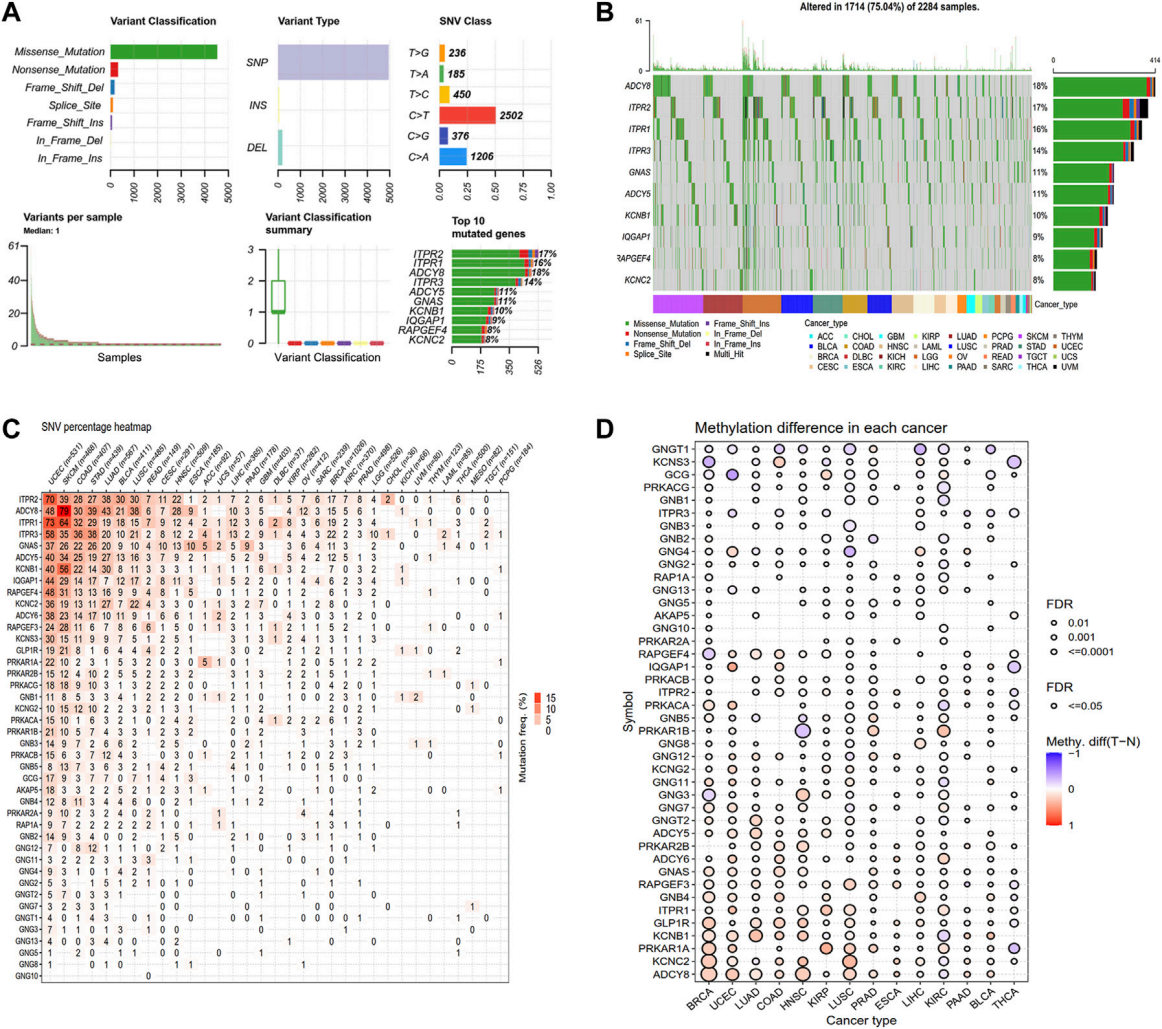
Genetic alteration landscape of GLP-1 signaling-related genes. **(A)** The SNV classes of related genes analyzed by GSCA platform. **(B)** Oncoplot depicting top 10 mutated genes. **(C)** Deleterious mutation frequency of related genes in each cancer type. **(D)** Differential analysis for methylation level among normal and tumor tissues in cancer types as indicated. The bubble denotes FDR ≤0.5, red bubble indicates hypermethylation and blue bubble indicates hypomethylation.

Next, we explored the genetic alterations of GLP-1 signaling-related genes in pan-cancer, including SNV class, variant classification, variant types and top 10 mutated genes. Results showed that single nucleotide mutations were mainly C > T, accounting for more than 50% and most of the gene mutations were missense mutations (Figure 2A). Among GLP-1 signaling-related genes, ADCY8 has the highest mutation frequency (18%) (Figure 2B). Furthermore, the frequencies of gene deleterious mutations in pan-cancer varied from each other. In general, ADCY8 in SKCM (79%) had the highest proportion of total deleterious mutations, followed by ITPR1 in UCEC (73%) (Figure 2C). As for methylation, GNGT1, KCNS3, GCG and PPKACG tended to be hypomethylated in pan-cancer while ADCY8 tended to be hypermethylated (Figure 2D).

**FIGURE 3:**
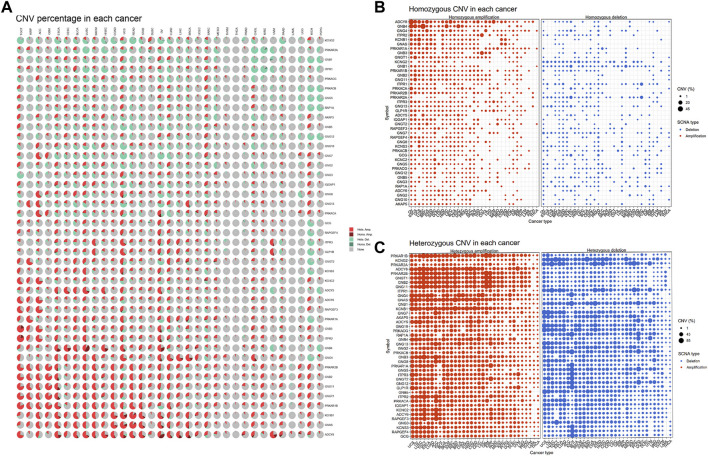
The CNV pattern of GLP-1 signaling-related genes. **(A)** Pie plot for CNV percentage of related genes in each cancer type. **(B,C)** The profile showing the percentage of homozygous CNV **(B)** and heterozygous CNV **(C)** of related genes in pan-cancer. The dot size represents CNV percentage, the red dot represents genetic amplification and blue dot represents genetic deletion in **(B,C)**. SCNA: somatic copy number alterations.

We also analyzed the CNV of GLP-1 signaling-related genes, including homozygous amplification, homozygous deletion, heterozygous amplification, and heterozygous deletion (Figure 3A). Overall, the genes related to GLP-1 signaling were heterozygous mutations predominantly. In term of homozygous amplification analysis, the percentage of GNB4 in LUSC as well as ADCY8 and GNB4 in OV were greater than 23%. The highest homozygous deletion percentage was about 10%, were PRKAR2A in DLBC, ITPR1 and PRKAR2A in KIRC, respectively (Figure 3B). For heterozygous mutation analysis, amplification rate of genes such as ADCY8, GNB2, GNG11, GNGT1, PRKAR1B, PRKAR2B in ACC, BLCA, BRCA, ESCA, KICH, LUSC, OV, READ, SARC, SARC SKCM, TGCT, UCS were greater than 23%, while deletion rate of GCG, RAPGEF4 KCNS3, GLP-1R, GNG12, GNGT2, ITPR3, GNG5, PRKAR1A in KICH were greater than 43% (Figure 3C).

### 3.3 GLP-1 signaling scores are associated with prognosis of patients with cancer

**FIGURE 4:**
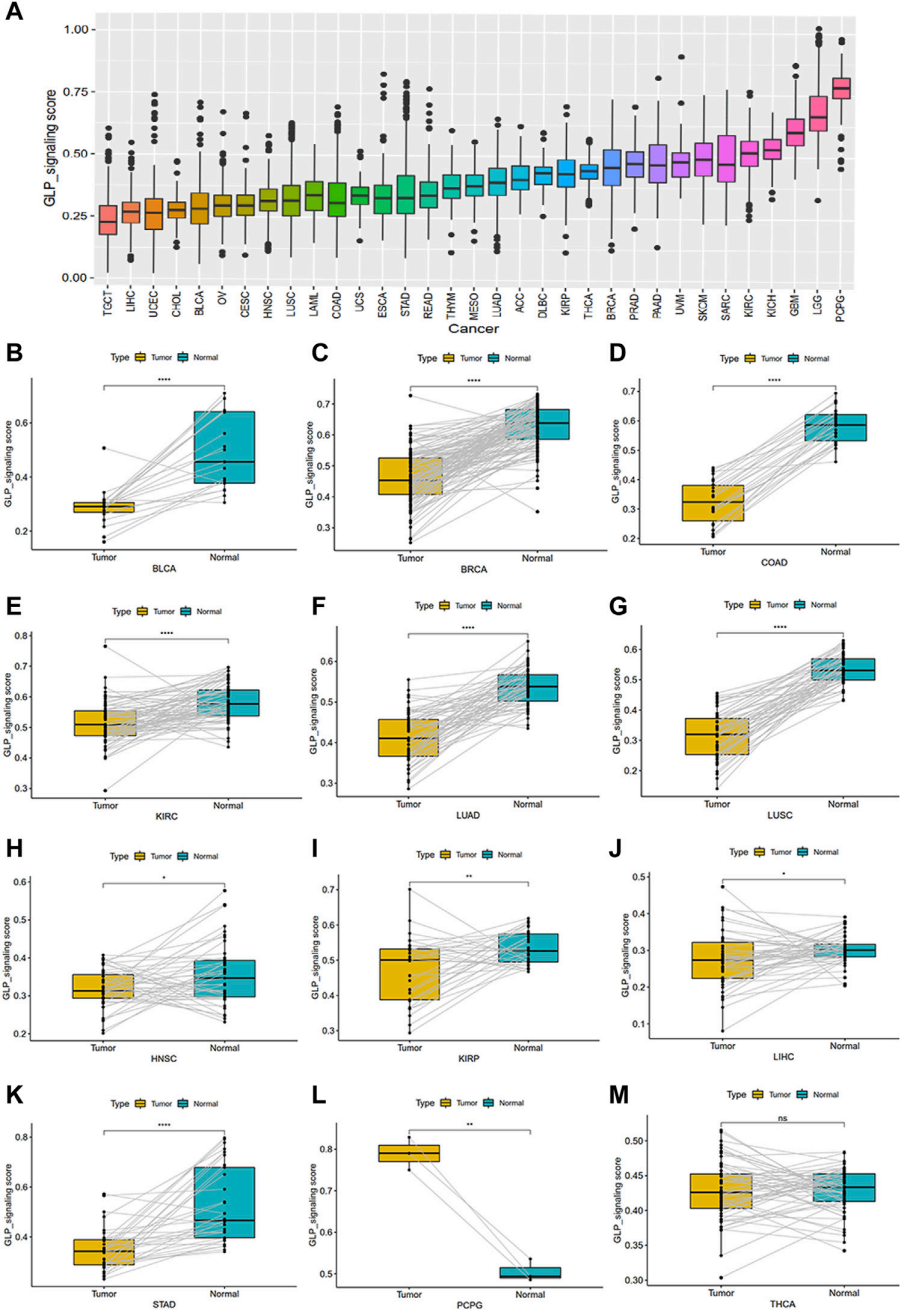
The estimated score of GLP-1 signaling and their differential analysis among samples in TCGA database. **(A)** The distribution of GLP-1 signaling score among 33 cancer types. **(B–M)** Differential analysis for GLP-1 signaling scores. Compared with paired normal tissues, scores were lower in BLCA, BRCA, COAD, KIRC, LUAD, LUSC, HNSC, KIRP, LIHC, STAD **(B–K)** and higher in PCPG **(L)**, while no significance was found in THCA **(M)**. ∗ *p* < 0.05; ∗∗ *p* < 0.01; ∗∗∗ *p* < 0.001; ∗∗∗∗ *p* < 0.0001; ns, not significant.

**FIGURE 5:**
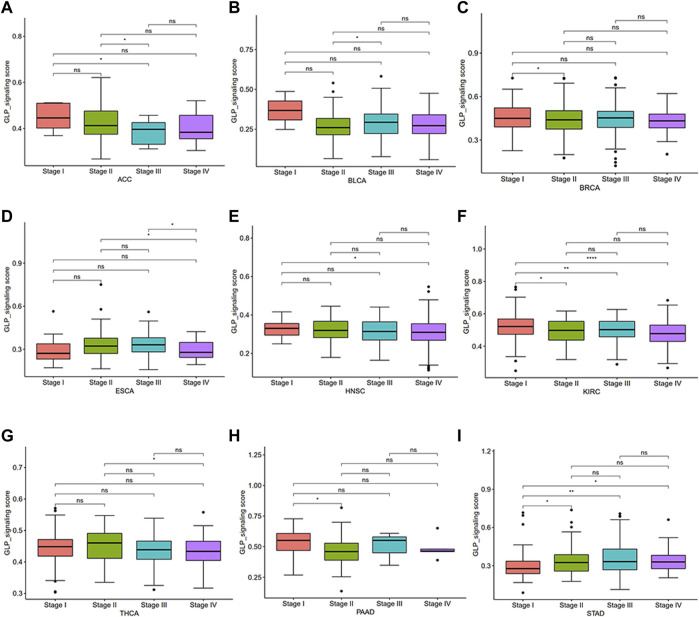
Differential analysis for GLP-1 signaling score among clinical stages in pan-cancer **(A–I)**. ∗ *p* < 0.05; ∗∗ *p* < 0.01; ∗∗∗ *p* < 0.001; ∗∗∗∗ *p* < 0.0001; ns, not significant.

To investigate the overall role of GLP-1 signaling-related genes in the development of cancers, an estimated model of GLP-1 signaling score was constructed based on enrichment scores by ssGSEA (Figure 4A). Among the 33 types of tumors, the highest GLP-1 signaling score was in PCPG and the lowest score was in TGCT. We then assessed the scores between tumor and normal tissues. The results suggested that GLP-1 signaling scores were lower in majority of tumors compared with paired normal tissues, such as BLCA, BRCA, COAD, KIRC, LUAD, LUSC, HNSC, KIRP, LIHC, STAD (Figures 4B–K). In comparison, GLP-1 signaling scores were higher in PCPG (Figure 4L), while no significance was found in THCA (Figure 4M). We also evaluated the relationship between GLP-1 signaling score and clinical stage in pan-cancer, which showed that the score changed with tumor stage. For instance, with the progression of clinical stage, GLP-1 signaling score decreased in most cancers, including ACC, BLCA, BRCA, ESCA, HNSC, KIRC, THCA and PAAD (Figures 5A–H), while the opposite trend was observed in STAD (Figure 5I).

**FIGURE 6:**
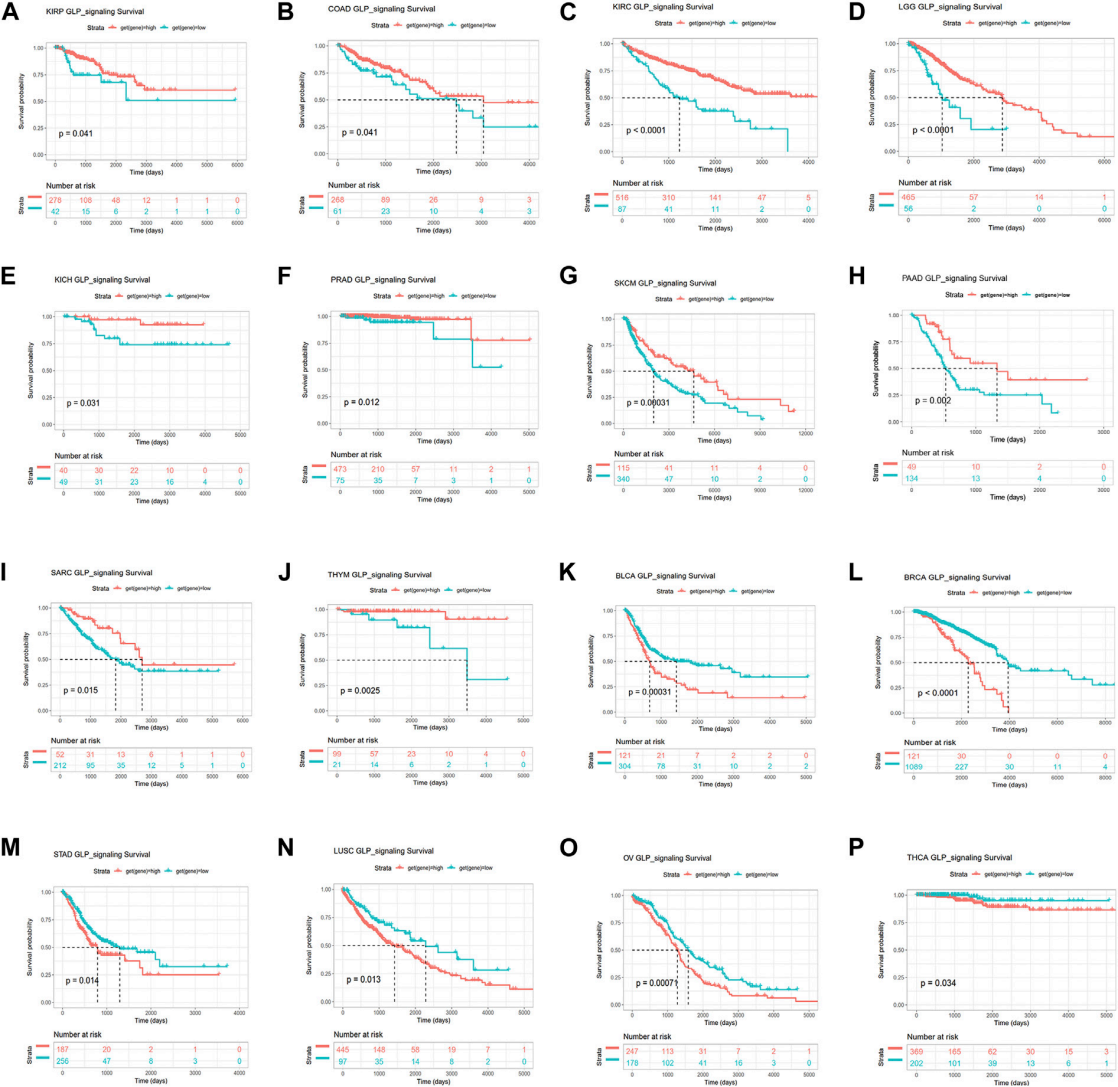
Kaplan-Meier overall survival analysis for high and low GLP-1 signaling score in TCGA cohort. The survival curves indicate higher GLP-1 signaling score is associated with better survival for KIRP, COAD, KIRC, LGG, KICH, PRAD, SKCM, PAAD, SARC and THYM **(A–J)**, and worse survival for BLCA, BRCA, STAD, LUSC, OV and THCA **(K–P)**.

**FIGURE 7:**
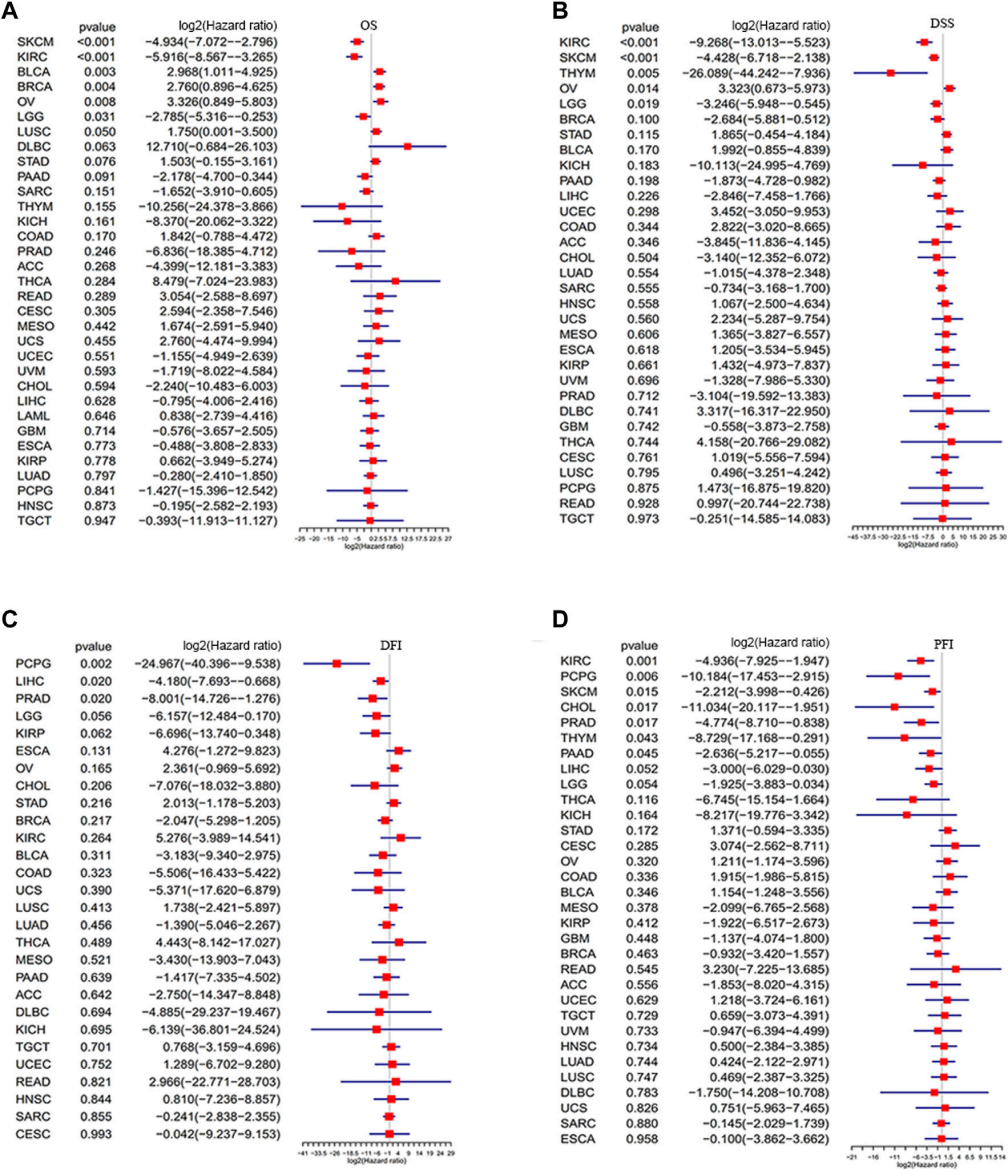
Forest plots depicting the Cox regression analysis for GLP-1 signaling score in pan-cancer **(A–D)**. OS: overall survival; DSS: disease specific survival; DFI: disease-free interval; PFI: progression free interval.

To further identify the prognostic significance of GLP-1 signaling-related genes in pan-cancer, Kaplan-Meier analyses with the best cut-off value and Univariate Cox regression analyses were performed in patients from TCGA database. The results of Kaplan-Meier OS analyses indicated that higher GLP-1 signaling score was associated with better survival for KIRP, COAD, KIRC, LGG, KICH, PRAD, SKCM, PAAD, SARC and THYM (Figures 6A–J), and worse survival for BLCA, BRCA, STAD, LUSC, OV and THCA (Figures 6K–P). In addition, results of univariate Cox regression analysis for OS showed that higher GLP-1 signaling score was associated with favorable prognosis for patients with SKCM, KIRC and LGG, while poor prognosis for patients with BLCA, BRCA and OV (Figure 7A). For DSS, higher GLP-1 signaling score was associated with favorable prognosis in KIRC, SKCM, THYM and LGG, while poor prognosis in OV (Figure 7B). For DFI, higher GLP-1 signaling score was associated with favorable prognosis in PCPG, LIHC and PRAD (Figure 7C). For PFI, higher GLP-1 signaling score was associated with favorable prognosis in KIRC, PCPG, SKCM, CHOL, PRAD, THYM and PAAD (Figure 7D).

### 3.4 GLP-1 signaling scores relate to immune signature

**FIGURE 8:**
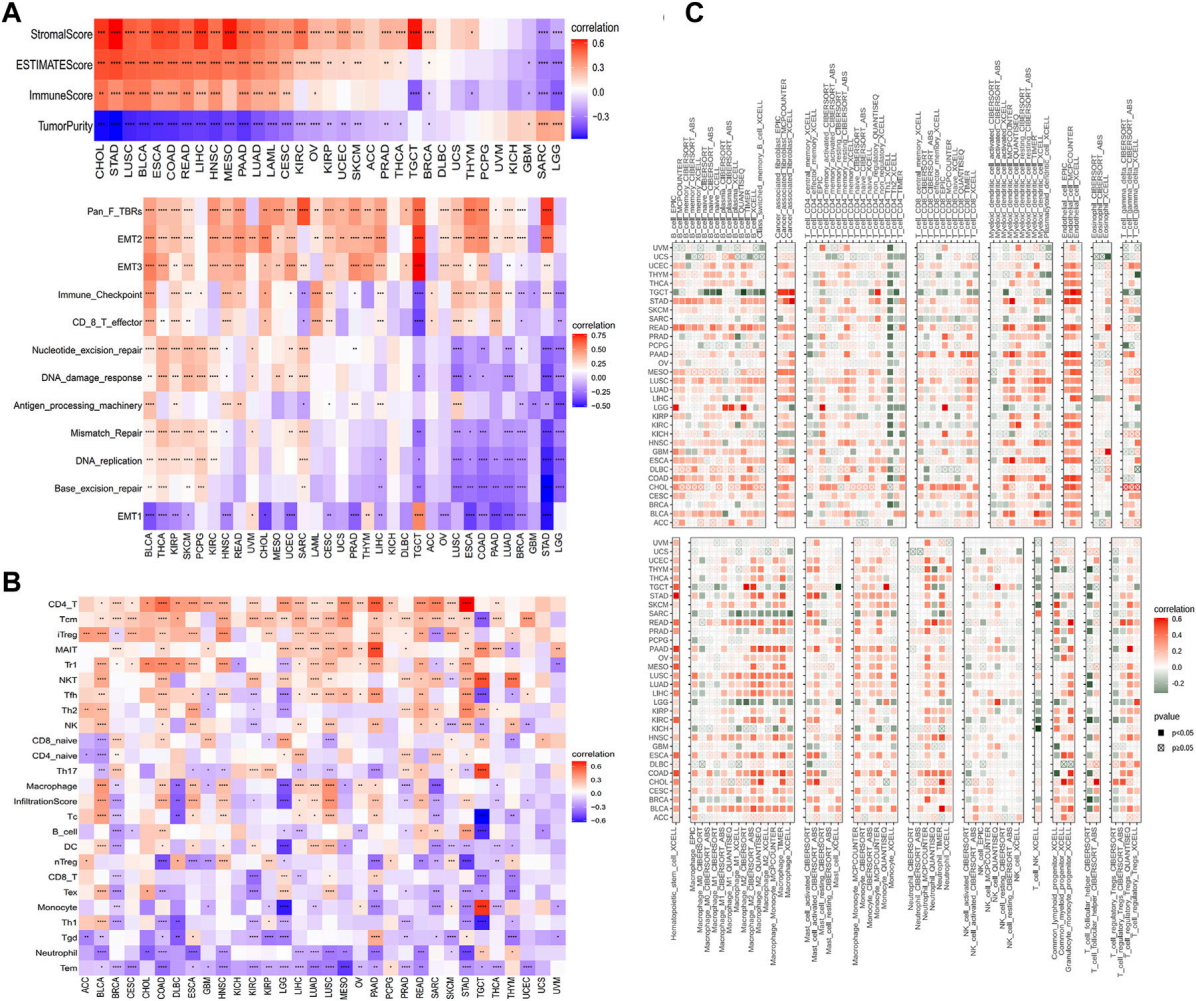
Correlations between GLP-1 signaling score and immune features. **(A)** Analysis of the tumor microenvironment based on GLP-1 signaling score. **(B,C)** Relationship between GLP-1 signaling score and immune cell infiltration based on ImmuCellAI database **(B)** and TIMER2 database **(C)**. The gradation of color denotes Pearson correlation coefficient. Red grid denotes positive correlation while blue or green grid denotes negative correlation. ∗ *p* < 0.05; ∗∗ *p* < 0.01; ∗∗∗ *p* < 0.001; ∗∗∗∗ *p* < 0.0001.

There are a wide range of cell types supporting cancer cells, such as fibroblasts, endothelial cells, macrophages, T cells, B cells, and so on. These cells in the TME cooperate or compete with each other to affect tumor growth, metastasis and immune evasion (de Visser and Joyce, 2023; Fang et al., 2023; Laumont and Nelson, 2023). To understand the association between GLP-1 signaling and TME, we estimated the stromal score, immune score and tumor purity of each patient in TCGA. The results indicated that GLP-1 signaling score was strongly correlated with TME (stromal and immune) scores in most cancer types (*p* < 0.05) (Figure 8A). Furthermore, GLP-1 signaling score was closely associated with TME-related pathways, including EMT, antigen processing machinery, CD8 T effector, immune checkpoint, DNA damage and mismatch repair in pan-cancer (Figure 8A). In addition, gene sets from Reactome Pathway Database were selected and GSEA was performed using the R package “clusterProfiler”. The results suggested that GLP-1 signaling was significantly associated with the enrichment of several immune-related pathways (innate immune system, adaptive immune system and cytokine signaling in immune system), such as in BLCA, CESC, HNSC, LIHC, LUAD and LUSC (adjust. *P* < 0.05) (Supplementary Figure S2).

Since immune cell infiltration is critical to TME and the response of immunotherapy, we estimated the relationship between GLP-1 signaling score and immune cell infiltration of samples in TCGA based on data from the ImmuCellAI database and the TIMER2 database. As shown in these results, positive associations were observed between GLP-1 signaling scores and many T cells, such as CD4^+^ T cells, central memory T cells (Tcm), natural killer (NK) cells, Follicular helper T cells (Tfh), in most cancer types. In contrast, the scores were negatively associated with neutrophils and monocytes. As for dendritic cells and macrophages, they were positively associated with GLP-1 signaling scores in BLCA, LUAD and LUSC, while negative associations were observed in BRCA, LGG and SARC (Figures 8B, C). Additionally, the GLP-1 signaling scores were closely related to immune-activating genes, MHC genes, chemokines and chemokine receptors in most types of cancer (Supplementary Figure S3), which indicated that the GLP-1 signaling is involved in immune response to tumor cells.

### 3.5 GLP-1 signaling score is associated with immunotherapy response in many cancer types

**FIGURE 9:**
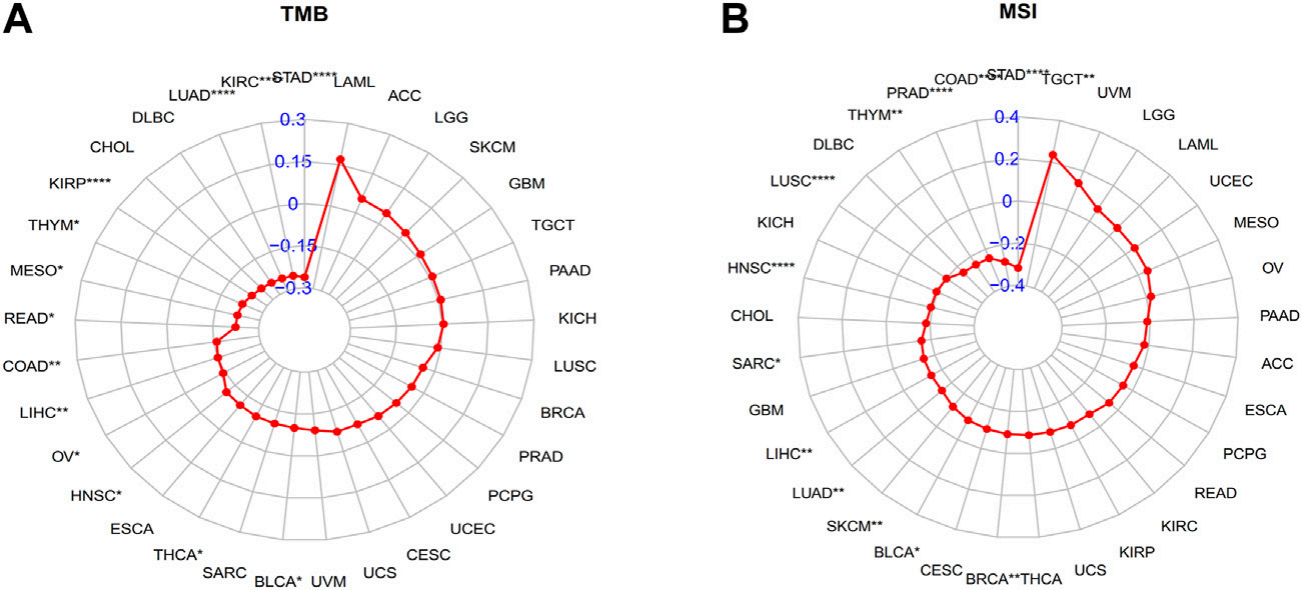
Radar plots depicting correlation between GLP-1 signaling score and TMB **(A)** and MSI **(B)** level. TMB: tumor mutation burden; MSI: microsatellite instability. ∗ *p* < 0.05; ∗∗ *p* < 0.01; ∗∗∗ *p* < 0.001; ∗∗∗∗ *p* < 0.0001.

Immunotherapy has become an important treatment for malignant tumors (André et al., 2020; Hong et al., 2020). A large body of evidence indicates that a high tumor mutation burden (TMB) and microsatellite instability (MSI) may be sensitive to tumor immunotherapy (Luchini et al., 2019; Huang et al., 2021). In our study, GLP-1 signaling score was negatively correlated with TMB in KIRP, THYM, MESO, READ, COAD, LIHC, OV, HNSC, THCA, BLCA, LUAD, KIRC, and STAD (Figure 9A). Similarly, GLP-1 signaling score was mainly negatively correlated with MSI, including LIHC, LUAD, SKCM, BLCA, BRCA, STAD, COAD, PRAD, THYM, LUSC, HNSC and SARC, while positively correlated with MSI in TGCT (Figure 9B).

**FIGURE 10:**
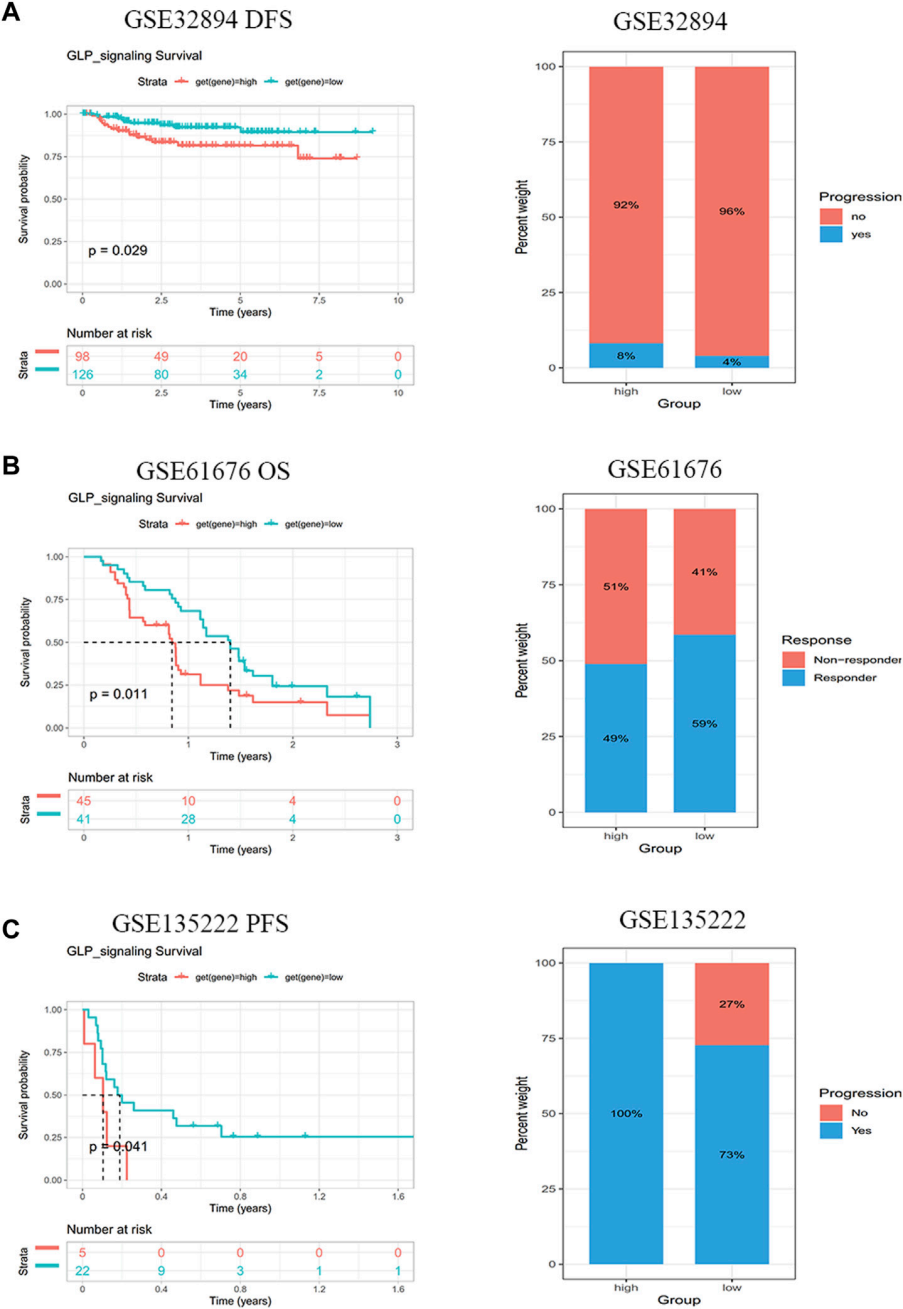
GLP-1 signaling score is connected with immunotherapy response. Patients with lower GLP-1 signaling score had better clinical outcomes after immunotherapy in GSE32894 **(A)**, GSE61676 **(B)** and GSE135222 **(C)** cohorts from GEO database.

Based on above results, the patients with low GLP-1 signaling score may benefit from immune checkpoint inhibitor treatment. To verify the effect of GLP-1 signaling score on prognosis of patients with immunotherapy, we collected and analyzed three datasets from GEO database (GSE32894, GSE61676 and GSE135222). The results indicated that a lower GLP-1 signaling score was related to more effective response and a better clinical outcome in patients with urothelial carcinoma (GSE61676) and non-small cell lung cancer (GSE61676 and GSE135222) (Figure 10).

### 3.6 Validation of the expression of GLP-1 signaling-related genes in CRC tissues

**FIGURE 11:**
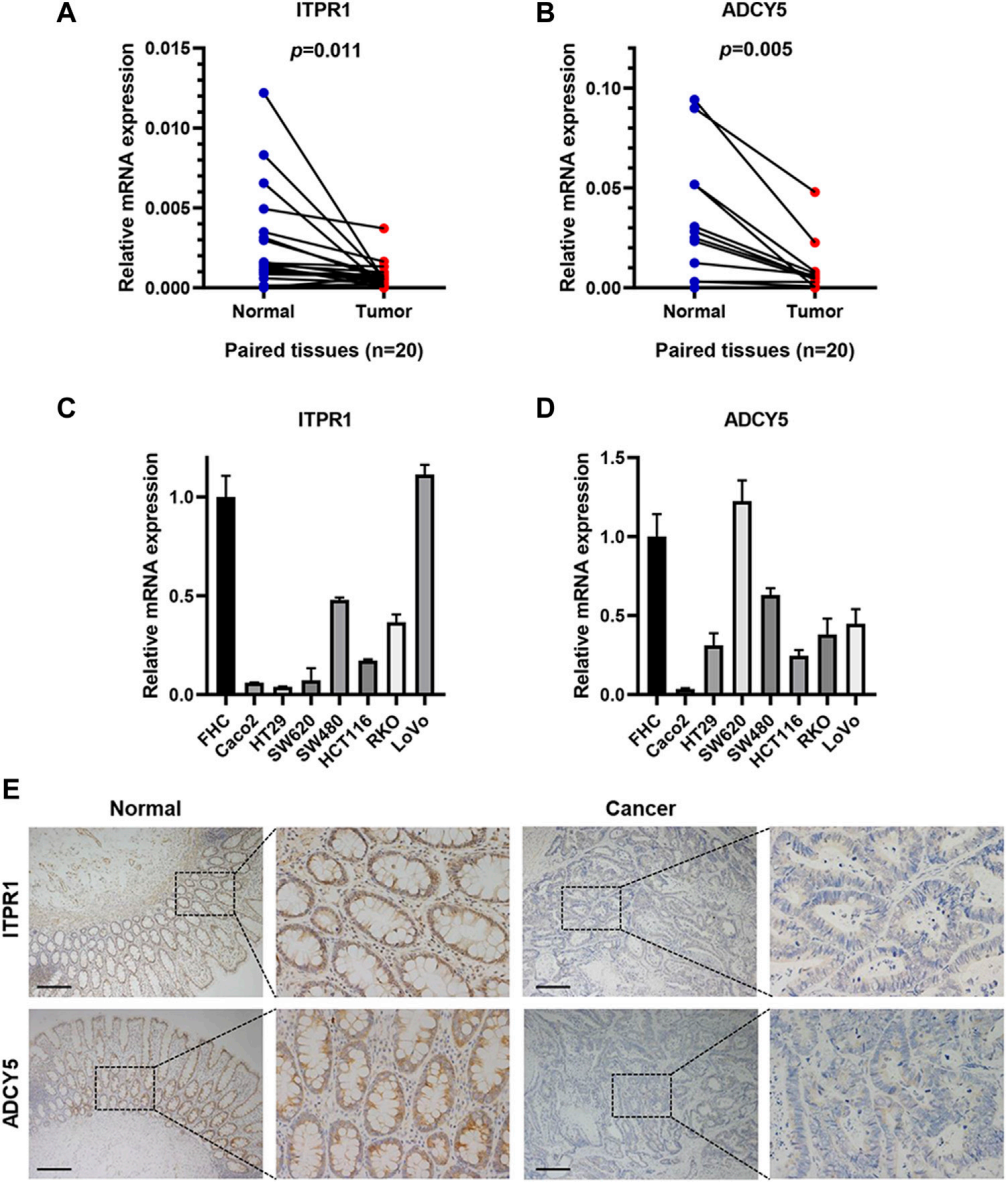
ITPR1 and ADCY5 expression in colorectal cancer. **(A,B)** qRT-PCR analyses showed mRNA expression of ITPR1 **(A)** and ADCY5 **(B)** in CRC tissues and paired normal tissues (n = 20). **(C,D)** ITPR1 **(C)** and ADCY5 **(D)** mRNA expression in CRC cell lines and normal colorectal mucosal cells. **(E)** IHC analyses demonstrated the expression of ITPR1 and ADCY5 in CRC tissues and adjacent normal mucosa. Representative images of IHC staining are showed (n = 5). Scale bar represents 200 μm.

Subsequently, we performed protein-protein interaction network analysis among 42 genes related to GLP-1 signaling (Supplementary Figure S1) and selected top 10 genes with highest node-degree for further validation in CRC tissues (Supplementary Table S2). Among the 10 genes, the mRNA expression of ITPR1 and ADCY5 in CRC tissues was prominently lower than normal tissues (n = 20) (Figures 11A, B; Supplementary Figure S4). Additionally, compared with normal colorectal mucosal cells, the mRNA expression of ITPR1 and ADCY5 were reduced in majority CRC cell lines (Figures 11C, D). IHC staining indicated that ITPR1 and ADCY5 expression were decreased in tumor tissues than normal tissues (Figure 11E). These results suggested that ITPR1 and ADCY5 may act as cancer suppressing factors.

### 3.7 GLP-1 receptor agonist semaglutide inhibits the migration capacity of CRC cells

**FIGURE 12:**
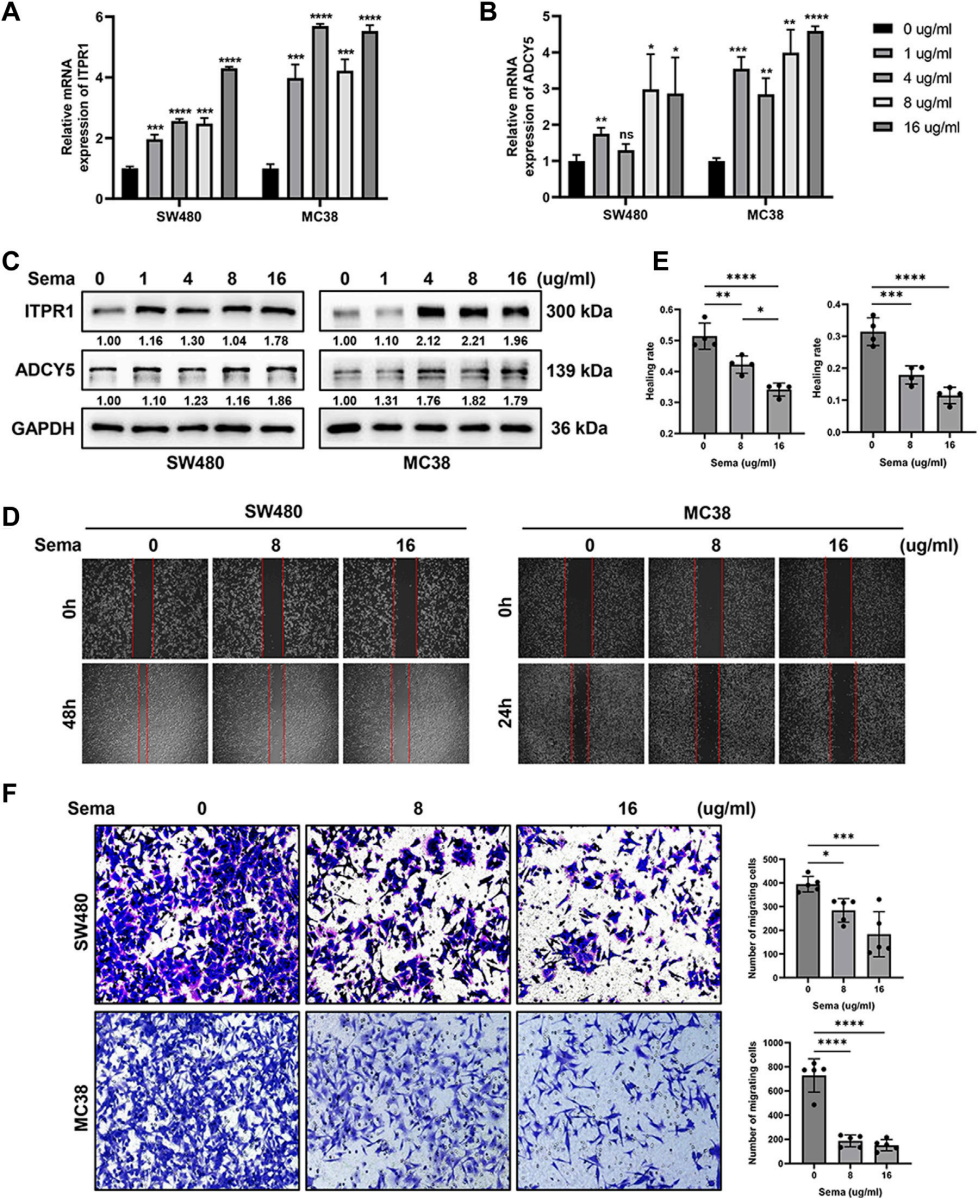
GLP-1 receptor agonist semaglutide promotes the expression of ITPR1 and ADCY5 and inhibits the migration of CRC cells. **(A,B)** qRT-PCR analyses indicated that semaglutide promoted ITPR1 **(A)** and ADCY5 **(B)** expression in a dose-dependent manner in CRC cells. **(C)** Western blot assays showed that ITPR1 and ADCY5 expression increase in a dose-dependent manner after incubation with different concentration of semaglutide. **(D)** Wound-healing assays revealed the effect of different concentration of semaglutide in SW480 (left) and MC38 (right) cells. **(E)** Quantitative analyses of healing rate of SW480 and MC38 cells. **(F)** Migration assays of colorectal cancer cells after incubation with semaglutide. Sema: semaglutide. *p < 0.05, **p < 0.01, ***p < 0.001, ****p < 0.0001.

To further explore the function of GLP-1 receptor agonist, we treated CRC cell lines SW480 and MC38 with semaglutide and detected the expression of ITPR1 and ADCY5. qRT-PCR and western blot assays showed that the expression of ITPR1 and ADCY5 increased in CRC cells in a dose-dependent manner after incubation with semaglutide (Figures 12A–C). In addition, the migrating cells were significantly reduced after semaglutide treatment (Figures 12D–F), suggesting that semaglutide may be a promising strategy to suppress the progression of CRC.

## 4 Discussion

Obesity and diabetes are known to be associated with an increased risk of malignancies such as endometrial cancer, colorectal cancer and postmenopausal breast cancer (Lega and Lipscombe, 2020; Kim and Scherer, 2021). Previous studies have shown that weight loss is correlated with a reduced risk of obesity-related cancers. In an observational study, multivariate analyses showed that postmenopausal women who lost weight had a significantly lower risk of endometrial cancer compared with women with stable weight, and this association was strongest among obese women who intentionally lost weight (Luo et al., 2017).

As an emerging treatment for antidiabetic, GLP-1 receptor agonists have satisfactory effect on stable glucose control (Tamborlane et al., 2019), and play a crucial role in weight loss, appetite inhibition and gastric emptying in obese patients (Nadolsky and Agarwal, 2021; Rubino et al., 2022). Hence, GLP-1 receptor agonists are more and more widely used in clinical practice. However, whether the GLP-1 receptor agonists have protective effects or hazard effects for pan-cancer is still unclear. Therefore, this study preliminarily explored the effect of genes related to GLP-1 signaling in pan-cancer.

We first found the differential expression of GLP-1 signaling-related genes in pan-cancer and the genes tended to be positively correlated with each other, suggesting that they may cooperate and function as a whole. Therefore, we constructed a score model based on the enrichment of genes related to GLP-1 signaling. Then we observed that GLP-1 signaling score of tumor tissues was lower than that of normal tissues in most cancers. Moreover, with the progression of the tumor, GLP-1 signaling score further decreased. This is consistent with previous studies. For example, GNG7 is downregulated in a variety of malignancies, and overexpression of GNG7 can inhibit cell proliferation and increase cell death, indicating that GNG7 may be a protective factor in cancer (Liu et al., 2016). In addition, previous studies indicate that the expression of ADCY5 is reduced and contributes to the progression of breast cancer (Rebbeck et al., 2022; Jung et al., 2023).

We also found that the survival outcomes of patients with high GLP-1 signaling score were generally better than those with low score, suggesting that it was a protective factor in these cancers, especially in KIRC, SKCM, LGG, ESCA. For instance, the survival rate of patients with high GNG7 expression is significantly higher than that of patients with low GNG7 expression in esophageal cancer, and low GNG7 expression leads to a deeper invasion *in vitro* and *in vivo* (Ohta et al., 2008). Additionally, GNG7 is downregulated in clear cell renal cell carcinoma and positively correlates with overall survival of patients (Xu et al., 2019a), implying a protective role of GNG7. However, there exists exceptions. GNG4 is significantly upregulated in primary gastric cancer and liver metastasis. High levels of GNG4 in primary cancer tissues are associated with short overall survival and high possibility of liver metastasis of gastric cancer (Tanaka et al., 2021), suggesting an oncogenic effect of GNG4.

We further analyzed the variation and methylation of GLP-1 signaling-related genes, which suggesting ADCY8 had the highest mutation rate in single nucleotide mutations and was inclined to be hypermethylated in pan-cancer. Previous studies showed that the methylation of ADCY8 promoter in cervical smears was correlated with worsening cytological grade (Shen-Gunther et al., 2016; Shen-Gunther et al., 2020). Another study demonstrated that GNG7 suppression presented with worse survival outcomes, which may result from promoter hypermethylation in esophageal cancer (Ohta et al., 2008). These findings indicate that epigenetic changes of GLP-1 signaling-related genes are associated with tumor progression.

GLP-1 contributes to anti-tumor immunity in some cancer types (Baldassano et al., 2017; Lu et al., 2021). In the analysis of immune cell infiltration, we found strong associations between GLP-1 signaling score and the infiltration of various immune cells such as CD4^+^ T cells, NK cells, neutrophils, dendritic cells and macrophages. As an indispensable member of anti-tumor immunity, CD4^+^ T cells promote anti-tumor immunity by assisting CD8 effector T cells or directly eliminate tumor cells by acting as cytotoxic T cells (Oh and Fong, 2021). Macrophages have important effect on inducing tumor cell apoptosis through phagocytosis or production of soluble factors as well as regulating tumor progression (Zhang and Zhang, 2020; Mao X. et al., 2021). It is well known that dendritic cells have functions such as antigen acquisition and presentation, induction of immunity, and play an important role in initiating immune responses and immune surveillance, making them ideal candidates for cancer immunotherapy (Sabado et al., 2017). A study shows that targeting WNT2 secreted by tumor-associated fibroblasts can restore dendritic cell-mediated antitumor immunity, suggesting its important role in anti-tumor immunity (Huang et al., 2022). GLP-1 analogue ameliorates the function of NK cells in people with obesity and activates NK cell-mediated anti-tumor responses in hepatocellular carcinoma (Lu et al., 2021; De Barra et al., 2023). The above information indicates that GLP-1 signaling-related genes may play a key role in anti-tumor immunity in pan-cancer and have an important impact on tumor cell elimination.

Notably, we also found a negatively correlation between GLP-1 signaling score and TMB/MSI in LIHC, LUAD, BLCA, HNSC, STAD, BLCA, COAD and so on, demonstrating there may be high microsatellite instability or tumor mutation burden in these tumors, thus antitumor immunity is more likely to be activated. In line with above findings, our results showed that a lower GLP-1 signaling score was related to a better clinical outcome in patients with urothelial carcinoma and non-small cell lung cancer after immunotherapeutic treatment. This study suggests that the patients with low GLP-1 signaling score may benefit from immune checkpoint inhibitor treatment in some cancers.

We further validated the value of GLP-1 signaling-related genes in tumors, which found that ITPR1 and ADCY5 were low expressed in CRC tissues. We further treated CRC cells with semaglutide and found that the expression of ITPR1 and ADCY5 was activated, along with inhibition of tumor cell migration. These results indicated a potential protective role of GLP-1 receptor agonist, maybe via ITPR1 and ADCY5. Previous study also shows that upregulation of ITPR1 expression enhances autophagy, thereby sensitizes paclitaxel cytotoxicity (Xu et al., 2019b), suggesting a favorable prognosis in human cancer.

## 5 Conclusion

This study presents a comprehensive assessment of the expression profile and prognostic value of GLP-1 signaling-related genes, revealing its protective role in most cancers. Moreover, we elaborate the immune infiltration landscape of GLP-1 signaling score in pan-cancer and reveal its potential immunotherapeutic values. Notably, we validate the expression of GLP-1 signaling-related genes ITPR1 and ADCY5, as well as the potential protective role of GLP-1 receptor agonist, suggesting a promising strategy for the treatment of CRC.

## Data Availability

The original contributions presented in the study are included in the article/Supplementary Material, further inquiries can be directed to the corresponding authors.
