# Supplementary material for: Comprehensively prognostic and immunological analyses of GLP-1 signaling-related genes in pan-cancer and validation in colorectal cancer
Source: Front Pharmacol. 2024 Jul 22;15:1387243. doi: 10.3389/fphar.2024.1387243 (PMC11298396; doi:10.3389/fphar.2024.1387243)
Supplement: Supplementary file 1 [file DataSheet1.docx]

Supplementary Material

# Supplementary Figures and Tables

## Supplementary Figures


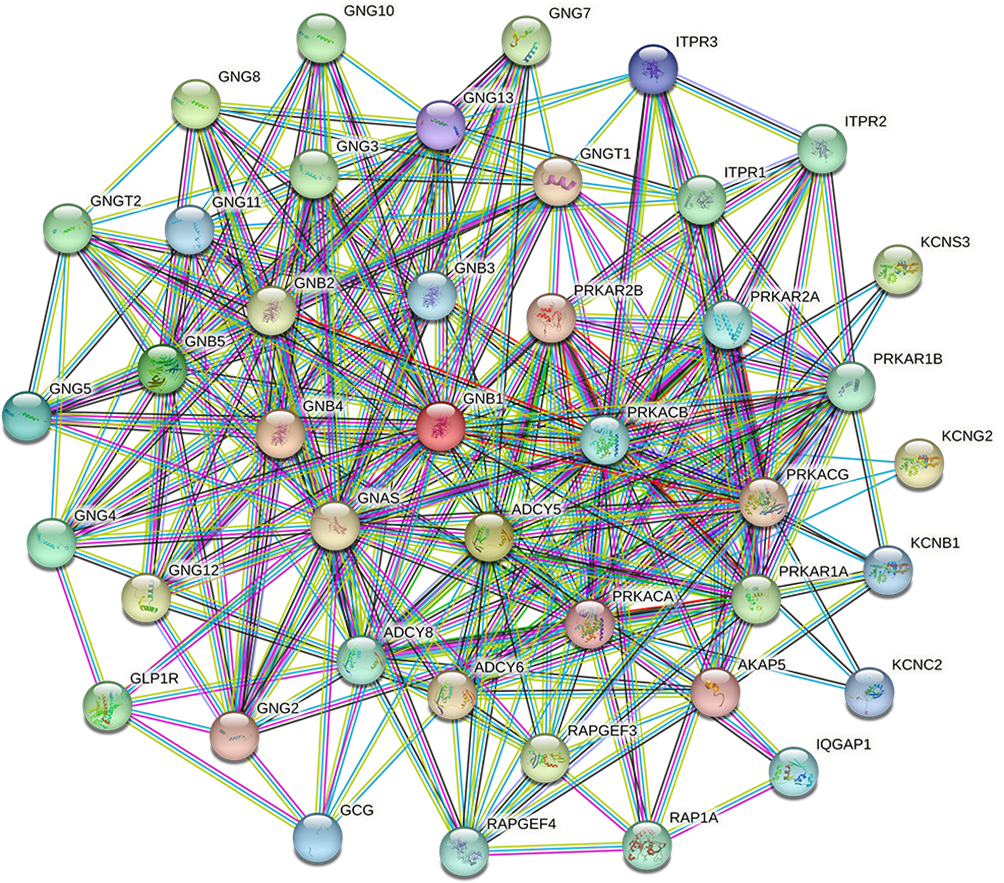


**Supplementary Figure S1** Protein–protein interaction network of 42 genes related to GLP-1 signaling.


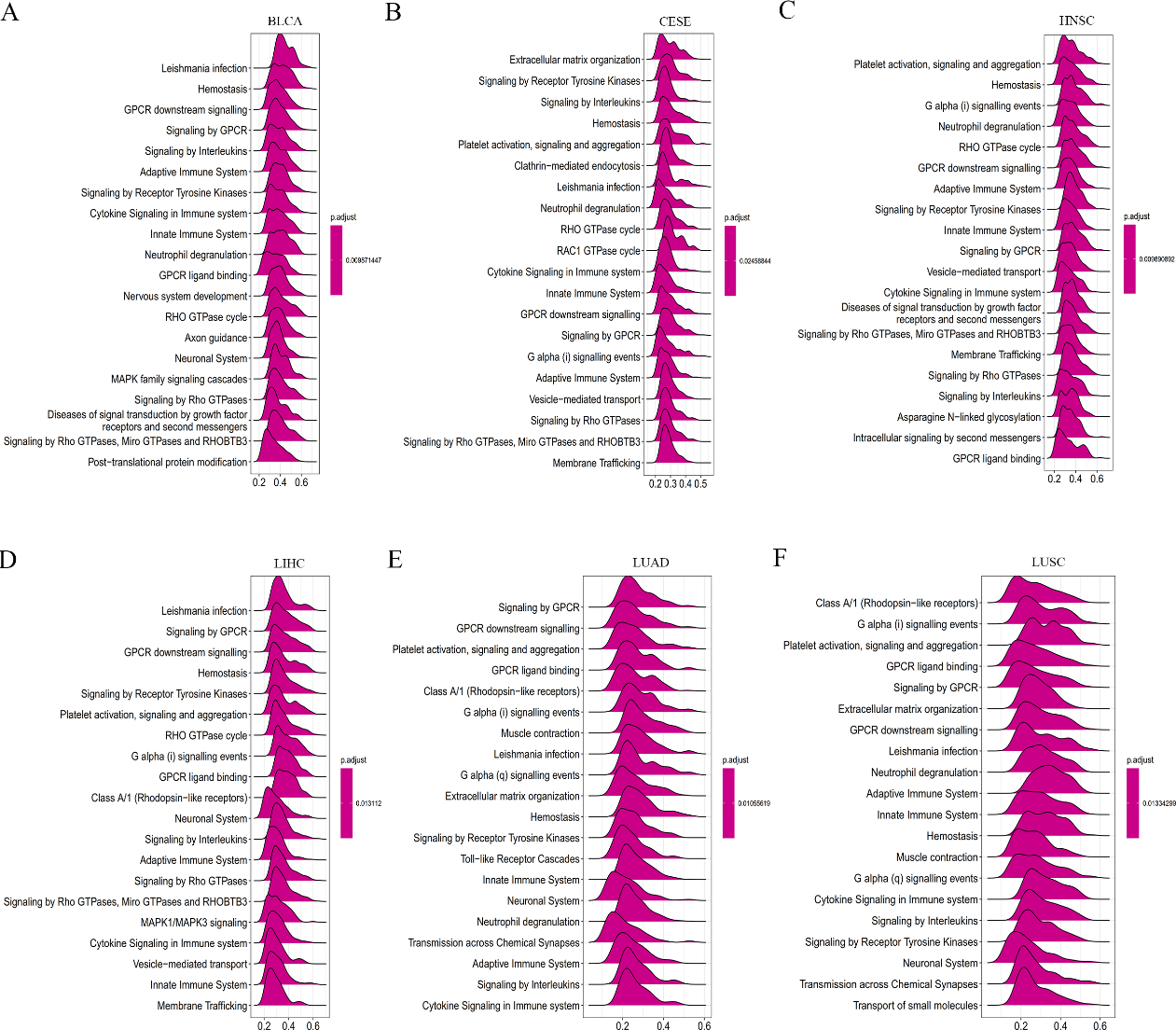


**Supplementary Figure S2** Gene set enrichment analysis indicating GLP-1 signaling associated with Reactome pathways in BLCA (**A**), CESC (**B**), HNSC (**C**), LIHC (**D**), LUAD (**E**) and LUSC (**F**).


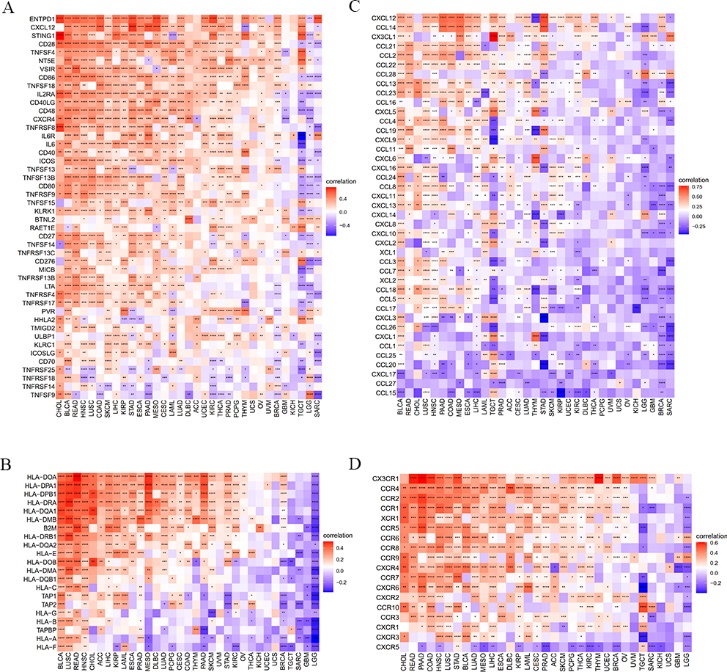


**Supplementary Figure S3** Correlations between the GLP-1 signaling score and immune-activating genes (**A**), MHC genes (**B**), chemokines (**C**) and chemokine receptors (**D**) based on Pearson analysis in each cancer type. ∗ *p* < 0.05; ∗∗ *p* < 0.01; ∗∗∗ *p* < 0.001; ∗∗∗∗ *p* < 0.0001.


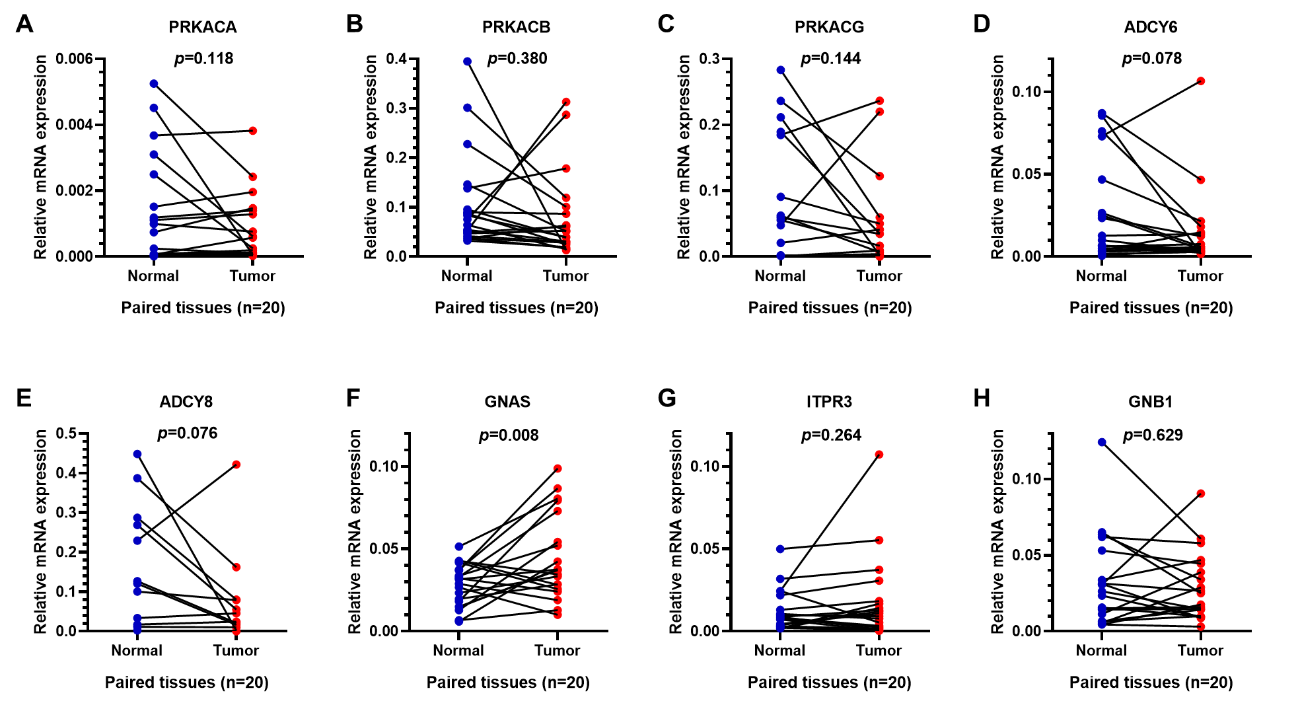


**Supplementary Figure S4** qRT-PCR analyses showed mRNA expression of genes related to GLP-1 signaling in CRC tissues and paired normal tissues (n=20).

## Supplementary Tables

**Supplementary Table S1** 33 types of cancer in TGCA

| Abbreviation | Full name | | |
| --- | --- | --- | --- |
| ACC | adrenocortical carcinoma | | |
| BLCA | bladder urothelial carcinoma | | |
| BRCA | breast invasive carcinoma | | |
| CESC | cervical squamous cell carcinoma and endocervical adenocarcinoma | | |
| CHOL | cholangiocarcinoma | |  |
| COAD | colon adenocarcinoma | | |
| DLBC | difuse large B-cell lymphoma | | |
| ESCA | oesophageal carcinoma | | |
| GBM | glioblastoma multiforme | | |
| HNSC | head and neck squamous cell carcinoma | | |
| KICH | kidney chromophobe | | |
| KIRC | kidney renal clear cell carcinoma | | |
| KIRP | kidney renal papillary cell carcinoma | | |
| LAML | acute myeloid leukaemia | | |
| LGG | lower-grade glioma | |  |
| LIHC | liver hepatocellular carcinoma | | |
| LUAD | lung adenocarcinoma | | |
| LUSC | lung squamous cell carcinoma | | |
| MESO | mesothelioma | |  |
| OV | ovarian serous cystadenocarcinoma | | |
| PAAD | pancreatic adenocarcinoma | | |
| PCPG | pheochromocytoma and paraganglioma | | |
| PRAD | prostate adenocarcinoma | | |
| READ | rectum adenocarcinoma | | |
| SARC | sarcoma |  |  |
| SKCM | skin cutaneous melanoma | | |
| STAD | stomach adenocarcinoma | | |
| TGCT | testicular germ cell tumours | | |
| THCA | thyroid carcinoma | |  |
| THYM | thymomat | |  |
| UCEC | uterine Corpus Endometrial Carcinoma | | |
| UCS | uterine carcinosarcoma | | |
| UVM | uveal melanoma | |  |

**Supplementary Table S2** GLP-1 signaling-related genes_string node degrees

| #node | identifier | node_degree |
| --- | --- | --- |
| PRKACA | 9606.ENSP00000309591 | 41 |
| PRKACB | 9606.ENSP00000359719 | 41 |
| PRKACG | 9606.ENSP00000366488 | 41 |
| ADCY6 | 9606.ENSP00000311405 | 34 |
| ADCY5 | 9606.ENSP00000419361 | 33 |
| ADCY8 | 9606.ENSP00000286355 | 33 |
| GNAS | 9606.ENSP00000360141 | 33 |
| ITPR1 | 9606.ENSP00000306253 | 30 |
| ITPR3 | 9606.ENSP00000363435 | 30 |
| GNB1 | 9606.ENSP00000367872 | 29 |
| ITPR2 | 9606.ENSP00000370744 | 29 |
| GNB2 | 9606.ENSP00000305260 | 28 |
| GNB3 | 9606.ENSP00000229264 | 28 |
| GNB4 | 9606.ENSP00000232564 | 28 |
| GNB5 | 9606.ENSP00000261837 | 28 |
| GNG10 | 9606.ENSP00000363411 | 28 |
| GNG11 | 9606.ENSP00000248564 | 28 |
| GNG12 | 9606.ENSP00000360021 | 28 |
| GNG13 | 9606.ENSP00000248150 | 28 |
| GNG2 | 9606.ENSP00000334448 | 28 |
| GNG3 | 9606.ENSP00000294117 | 28 |
| GNG4 | 9606.ENSP00000398629 | 28 |
| GNG5 | 9606.ENSP00000359675 | 28 |
| GNG7 | 9606.ENSP00000371594 | 28 |
| GNG8 | 9606.ENSP00000300873 | 28 |
| GNGT1 | 9606.ENSP00000248572 | 28 |
| GNGT2 | 9606.ENSP00000421710 | 28 |
| GCG | 9606.ENSP00000387662 | 27 |
| GLP1R | 9606.ENSP00000362353 | 27 |
| IQGAP1 | 9606.ENSP00000268182 | 22 |
| AKAP5 | 9606.ENSP00000378207 | 21 |
| PRKAR1A | 9606.ENSP00000376475 | 21 |
| PRKAR1B | 9606.ENSP00000385749 | 21 |
| PRKAR2A | 9606.ENSP00000265563 | 21 |
| PRKAR2B | 9606.ENSP00000265717 | 21 |
| RAPGEF3 | 9606.ENSP00000395708 | 19 |
| RAPGEF4 | 9606.ENSP00000380271 | 19 |
| KCNB1 | 9606.ENSP00000360806 | 11 |
| KCNC2 | 9606.ENSP00000449253 | 10 |
| KCNS3 | 9606.ENSP00000385968 | 10 |
| KCNG2 | 9606.ENSP00000315654 | 9 |
| RAP1A | 9606.ENSP00000358723 | 7 |

**Supplementary Table S3** Primers used in this study

| Gene | Forward (5' to 3') | Reverse (5' to 3') |
| --- | --- | --- |
| Homo sapiens ACTB | CCTGGCACCCAGCACAAT | GGGCCGGACTCGTCATAC |
| Homo sapiens ITPR1 | GGAGTTTCAGCCCTCAGTGG | TCAGCAGGAGAAACCGGAAC |
| Homo sapiens ADCY5 | AGTGTGTGGCGGTCATGTT | CTGCCGATGGTCTTGATCTT |
| Homo sapiens PRKACA | CAGCGGCAGAGATCTTGGG | TGTTCTGAGCGGGACTTTCC |
| Homo sapiens PRKACB | CCATGCACGGTTCTATGCAG | GTCTGTGACCTGGATATAGCCTT |
| Homo sapiens PRKACG | CGACTTTCCGTTCCTCGTCA | GCGAGTGTAGGTACTGGACG |
| Homo sapiens ADCY6 | GCTCATGGTGGTGTGTAACC | GCGTGTAGGCGATGTAGACAAA |
| Homo sapiens ADCY8 | TGCCCCTAGCAACTCGGAT | CCAGGCTCAAGTGTAGGACC |
| Homo sapiens GNAS | TGCCTCGGGAACAGTAAGAC | GCCGCCCTCTCCATTAAAC |
| Homo sapiens ITPR3 | CCAAGCAGACTAAGCAGGACA | ACACTGCCATACTTCACGACA |
| Homo sapiens GNB1 | TGCCTCGCAGGATGGTAAAC | CCGCAGGCCACATAGTTCC |
| Mus musculus Actb | GTGCTATGTTGCTCTAGACTTCG | ATGCCACAGGATTCCATACC |
| Mus musculus Itpr1 | ACGGATGCCAGGAGGAAATG | CAGGGGTGGACTTGGTTCAA |
| Mus musculus Adcy5 | GGGCGCAGTGAAAATATAGC | CTGCTTGGCGTTGATGTCTG |
